# Continuous *versus* bolus norepinephrine administration and arterial blood pressure stability during induction of general anaesthesia in high-risk noncardiac surgery patients: a randomised trial

**DOI:** 10.1016/j.bja.2025.06.025

**Published:** 2025-07-30

**Authors:** Christina Vokuhl, Karim Kouz, Moritz Flick, Linda Krause, Alina Kröker, Parisa Moll-Khosrawi, Christian Zöllner, Daniel I. Sessler, Bernd Saugel, Kristen K. Thomsen

**Affiliations:** 1 Department of Anesthesiology, Center of Anesthesiology and Intensive Care Medicine, University Medical Center Hamburg-Eppendorf, Hamburg, Germany; 2 Outcomes Research Consortium®, Houston, TX, USA; 3 Institute of Medical Biometry and Epidemiology, University Medical Center Hamburg-Eppendorf, Hamburg, Germany; 4 Center for Outcomes Research and Department of Anesthesiology, UTHealth, Houston, TX, USA

**Keywords:** anaesthesia induction, arterial pressure, cardiovascular dynamics, general anaesthesia, generalised average real variability, haemodynamic monitoring, vasopressor

## Abstract

**Background:**

Hypotension after induction of general anaesthesia is common in high-risk patients having noncardiac surgery. Anaesthesiologists often give manual boluses of vasopressors repeatedly to maintain blood pressure during induction of general anaesthesia, including the fast-acting vasopressor norepinephrine which has a short half-life. We tested the hypothesis that giving norepinephrine continuously during induction of general anaesthesia, compared with giving it as repeated manual boluses, improves blood pressure stability in high-risk noncardiac surgery patients.

**Methods:**

In this single-centre trial, 72 participants undergoing noncardiac surgery were randomised to continuous norepinephrine infusion or manual bolus norepinephrine administration during induction of general anaesthesia. Blood pressure was monitored continuously with an arterial catheter. The primary endpoint was blood pressure stability, quantified as the generalised average real variability of mean arterial pressure within 15 min after starting induction of general anaesthesia.

**Results:**

A total of 71 participants completed the study (mean [range] age: 66 [47-86] y; 48% female). The mean (standard deviation) generalised average real variability of mean arterial pressure was 19 (6) mm Hg min^−1^ in 36 participants assigned to continuous norepinephrine infusion, compared with 25 (7) mm Hg min^−1^ in 35 participants assigned to manual bolus norepinephrine administration (*P*<0.001).

**Conclusions:**

Giving norepinephrine continuously during induction of general anaesthesia, compared with giving it as repeated manual boluses, improved blood pressure stability in higher-risk individuals undergoing noncardiac surgery.

**Clinical trial registration:**

NCT05997303

**Editor’s key points**

• Hypotension after induction of general anaesthesia is common in high-risk patients having noncardiac surgery.
• The authors tested the hypothesis that giving norepinephrine continuously during induction of general anaesthesia, compared with manual boluses, improves blood pressure stability within 15 min after starting induction of general anaesthesia in high-risk noncardiac surgery patients.
• Blood pressure stability, quantified as the generalised average real variability of mean arterial pressure, was improved by giving norepinephrine continuously.
• The clinical impact of preserving blood pressure stability after induction of general anaesthesia remains unclear.

Hypotension after induction of general anaesthesia, referred to as postinduction hypotension, is common in patients having noncardiac surgery^1^, ^2^, ^3^ and is associated with acute kidney injury.^1^, ^2^, ^3^ Risk factors for postinduction hypotension include older age, higher ASA physical status, chronic arterial hypertension,^3^^,^^4^ and higher doses of vasodilating anaesthetic drugs.^5^^,^^6^

It is reasonable to assume that careful anaesthetic management will help maintain blood pressure stability and limit postinduction hypotension.^7^ Consistent with this assumption, continuous blood pressure monitoring with an arterial catheter during induction of general anaesthesia helps clinicians reduce postinduction hypotension^8^—and is thus recommended in noncardiac surgery patients at risk for hypotension-related complications.^9^^,^^10^ Besides continuous monitoring, anaesthesiologists routinely give vasopressors—such as norepinephrine—to maintain blood pressure. Norepinephrine is a fast-acting vasopressor with a short half-life,^11^ usually repeatedly given as manual boluses during induction of general anaesthesia. Given as a bolus, it effectively and rapidly increases blood pressure. However, boluses need to be frequently repeated and well-timed to ensure blood pressure stability. Continuous—instead of bolus—administration of norepinephrine during induction of general anaesthesia may thus help maintain blood pressure stability and reduce postinduction hypotension.

We therefore tested the primary hypothesis that giving norepinephrine continuously during induction of general anaesthesia—compared with giving it as repeated manual boluses—improves blood pressure stability in high-risk noncardiac surgery patients who have continuous blood pressure monitoring with an arterial catheter. We additionally investigated if giving norepinephrine continuously reduces the duration and severity of postinduction hypotension.

## Methods

### Trial design and setting

This single-centre randomised trial was conducted in the Department of Anesthesiology, Center of Anesthesiology and Intensive Care Medicine, University Medical Center Hamburg-Eppendorf, Hamburg, Germany, between September 3, 2023, and April 19, 2024. The trial was approved by the ethics committee (Ethikkommission der Ärztekammer Hamburg, Hamburg, Germany, registration number 2023-101053-BO-ff) on May 8, 2023, and registered at ClinicalTrials.gov (NCT05997303) on August 10, 2023. Participants provided written informed consent. We report the trial according to the Consolidated Standards of Reporting Trials (CONSORT) statement.^12^

### Inclusion criteria

We included participants scheduled for elective major noncardiac surgery with general anaesthesia who were at least 45 yr old, were classified as ASA physical status II to IV, were in normal sinus rhythm, and had a clinical indication for continuous intraarterial blood pressure monitoring with a radial arterial catheter.

### Exclusion criteria

We excluded participants who had a history of intracranial haemorrhage or intracranial aneurysms, were pregnant, or had a clinical indication for continuous norepinephrine infusion during induction of general anaesthesia (e.g. aortic valve stenosis or heart failure).

### Protocol

Before starting induction of general anaesthesia, all participants were equipped with routine monitoring, including electrocardiography, pulse oximetry, and intraarterial blood pressure monitoring with a radial artery catheter. We randomised participants in a 1:1 ratio without blocking or stratification to continuous norepinephrine infusion via a syringe infusion pump or to manual bolus norepinephrine administration. Group allocation was concealed until shortly before starting induction of general anaesthesia. Anaesthesiologists naturally were not blinded to group allocation, but participants were.

General anaesthesia was induced using sufentanil or remifentanil, propofol, and rocuronium and maintained with a continuous propofol infusion or inhaled sevoflurane. Types and doses of anaesthetic drugs were at the clinicians’ discretion. Anaesthesiologists strove to keep mean arterial pressure (MAP) above 65 mm Hg per institutional routine using balanced crystalloids given at the clinicians’ discretion and norepinephrine.

In participants assigned to continuous norepinephrine infusion, norepinephrine was given using a syringe infusion pump (Perfusor Space; B Braun, Melsungen, Germany) with a 50 mL syringe containing 3 mg norepinephrine (Arterenol; Cheplapharm, Greifswald, Germany; refrigerated) diluted in 0.9% normal saline. The solution was prepared by an anesthesiology nurse. Responsible anaesthesiologists adjusted the norepinephrine infusion rate as necessary and were free to give additional norepinephrine boluses via the syringe infusion pump if needed.

In patients assigned to manual bolus norepinephrine administration, norepinephrine was given manually as boluses from a 10 mL syringe containing 100 μg norepinephrine (Arterenol), prepared by an anesthesiology nurse, at the discretion of the treating anaesthesiologist.

### Blood pressure measurement

Blood pressure was measured continuously with the radial arterial catheter. We averaged beat-to-beat blood pressure values in non-overlapping 10-s windows to account for unequal heart rates and thus numbers of beat-to-beat blood pressure values per patient. Within these 10-s windows, single beat-to-beat blood pressure values were considered artifactual when (1) blood pressure values were documented as artifacts by trial personnel; (2) systolic arterial pressure (SAP) values were >280 mm Hg or <30 mm Hg; (3) SAP values were below diastolic arterial pressures plus 5 mm Hg; or (4) diastolic arterial pressures were >150 mm Hg or <10 mm Hg.

Artifactual beat-to-beat blood pressure values were replaced by the mean of the non-artifactual blood pressure values in the respective 10-s window. If all beat-to-beat blood pressure values in the 10-s window were artifacts, we replaced the missing mean value of this window using the mean of the two neighbouring 10-s windows. If all beat-to-beat blood pressure values in the first 10-s window were artifacts, we replaced the missing mean value of this first window using the mean of the second 10-s window. If all beat-to-beat blood pressure values in the first and second 10-s windows were artifacts, we replaced the missing mean values of these windows using the mean of the third 10-s window. Among 6392 10-s windows, only 31 windows had missing data (0.5%).

### Primary endpoint

The primary endpoint was blood pressure stability—quantified as the generalised average real variability of MAP (ARV-MAP)^13^ within 15 min after starting induction of general anaesthesia. The generalised ARV-MAP was calculated per individual participant by summing the absolute differences between the averaged MAP values of neighbouring 10-s windows, from the first to the last 10-s window, and dividing that sum by the total measurement time—in this trial, 15 min.^13^

### Secondary endpoints

We also assessed the following secondary endpoints:

1. Area under a MAP of 65 mm Hg within 15 min after starting induction of general anaesthesia
2. Areas under MAP values of 60, 50, and 40 mm Hg and above MAP values of 100, 110, 120, and 140 mm Hg
3. Cumulative durations of MAP values less than 65, 60, 50, and 40 mm Hg and higher than 100, 110, 120, and 140 mm Hg
4. Cumulative doses of norepinephrine, indexed to actual body weight

### *Post hoc* analysis

After peer review, we also calculated the generalised ARV-SAP. Additionally, we evaluated areas under SAP values of 90, 80, and 70 mm Hg and the cumulative durations of SAP values below these thresholds. We also analysed cardiac index and systemic vascular resistance index values over the 15-min trial period and calculated the generalised ARV of cardiac index and systemic vascular resistance index.

### Statistical analysis

The statistical analysis plan was included in the trial protocol and submitted to the ethics committee before the data were accessed. Patient, baseline, and clinical characteristics are reported separately for patients assigned to continuous norepinephrine infusion and patients assigned to manual bolus norepinephrine administration. Categorical data are presented as absolute number (percentage). Continuous data are presented as median (25th percentile, 75th percentile).

The primary endpoint, generalised ARV-MAP, was analysed using a two-sample two-sided *t*-test. We calculated the corresponding Cohen’s *d* to quantify the effect size, interpreting a Cohen’s *d* of <0.20 as negligible effect, 0.2 to <0.5 as a small effect, 0.5 to <0.8 as a medium effect, and ≥0.8 as a large effect. Continuous secondary endpoints were analysed using a Wilcoxon rank-sum test with continuity correction, and Cliff’s delta was calculated to quantify the respective effect size. We interpreted a Cliff’s delta of <0.15 as a negligible effect, 0.15 to <0.33 as a small effect, 0.33 to 0.47 as a medium effect, and >0.47 as a large effect. The generalised ARV-SAP—and the generalised ARV of cardiac index and systemic vascular resistance index—were analysed analogously to the primary endpoint. We used R Version 4.4.3 (R Foundation for Statistical Computing, Vienna, Austria) for statistical analyses.

### Sample size estimate

We estimated that a sample size of 32 participants per group (*n*=64 patients in total) was required to provide 91% power with a significance level (alpha) of 5% to detect a difference between the two groups of 5 mm Hg min^−1^ in generalised ARV-MAP when a standard deviation of 6 mm Hg min^−1^ was assumed for each group, using a two-sample two-sided *t*-test. The estimates for the difference and the standard deviation were based on previous studies.^8^^,^^13^ Anticipating a 10% drop-out rate, a total sample size of 72 participants (36 participants per group) was required. Sample size estimation was performed using PASS 2008 (NCSS, LLC, Kaysville, Utah, USA).

## Results

### Trial participants

**Fig 1:**
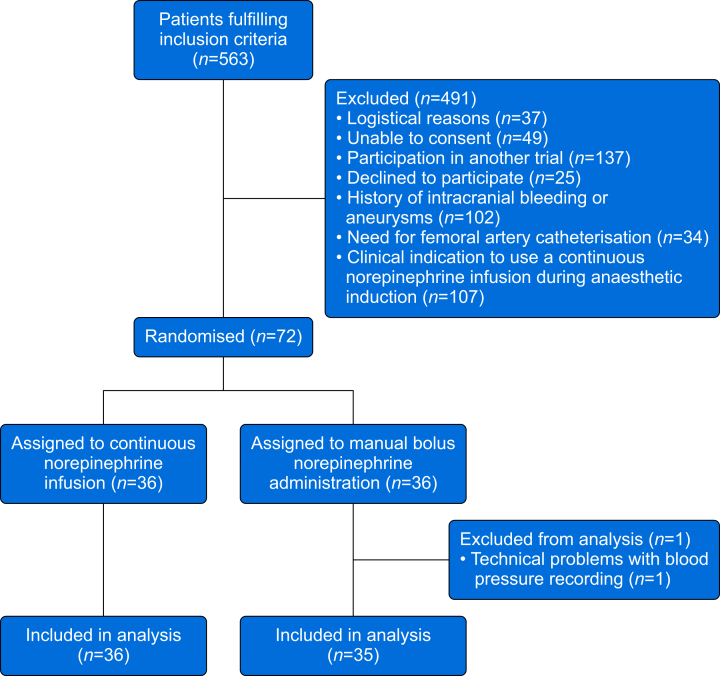
Trial flowchart. Flowchart illustrating patient screening, enrolment, randomisation, and reasons for exclusion.

A total of 72 participants were randomised but one participant was excluded because of technical problems with blood pressure recording (Fig. 1), leaving 36 participants assigned to continuous norepinephrine infusion and 35 participants assigned to manual bolus norepinephrine administration (Table 1).

**Table 1:** Participant characteristics and management during induction of general anaesthesia. Data are presented as mean (range or standard deviation), median (25th percentile, 75th percentile), or absolute number (percentage). Percentages may not sum up to 100% because of rounding.

Characteristic		Continuous norepinephrine infusion	Manual bolus norepinephrine administration
		(*n*=36)	(*n*=35)
Age (yr)		67 (55-86)	64 (47-81)
Height (cm)		174 (10)	172 (10)
Weight (kg)		80 (21)	79 (21)
Sex	Female	15 (42)	19 (54)
	Male	21 (58)	16 (46)
ASA physical status (2; 3)	2	20 (56)	21 (60)
	3	16 (44)	14 (40)
Chronic arterial hypertension		19 (53)	17 (49)
Chronic obstructive pulmonary disease		3 (8)	1 (3)
Diabetes mellitus		3 (8)	9 (26)
Chronic heart failure		0 (0)	0 (0)
Liver disease		0 (0)	0 (0)
Chronic kidney disease		2 (6)	2 (6)
Coronary artery disease		1 (3)	2 (6)
Cerebrovascular disease		2 (6)	1 (3)
Type of surgery			
General surgery		14 (39)	17 (49)
Gynaecological surgery		3 (8)	4 (11)
Neurosurgery		8 (22)	8 (23)
Urological surgery		9 (25)	6 (17)
Trauma surgery		2 (6)	0 (0)
Management during induction of general anaesthesia			
Epidural block		18 (50)	12 (34)
Propofol dose during induction (mg kg^−1^)		2.6 (2.0, 3.7)	2.7 (2.0, 3.8)
Sufentanil use		27 (75)	27 (77)
Sufentanil dose during induction (μg kg^−1^)		0.5 (0.4, 0.6)	0.5 (0.5, 0.6)
Remifentanil use		9 (25)	9 (26)
Remifentanil dose during induction (μg kg^−1^ min^−1^)		0.5 (0.4, 0.5)	0.5 (0.5, 0.5)
Cumulative volume of crystalloids (ml)		275 (200, 350)	250 (200, 435)

### Primary outcome

**Fig 2:**
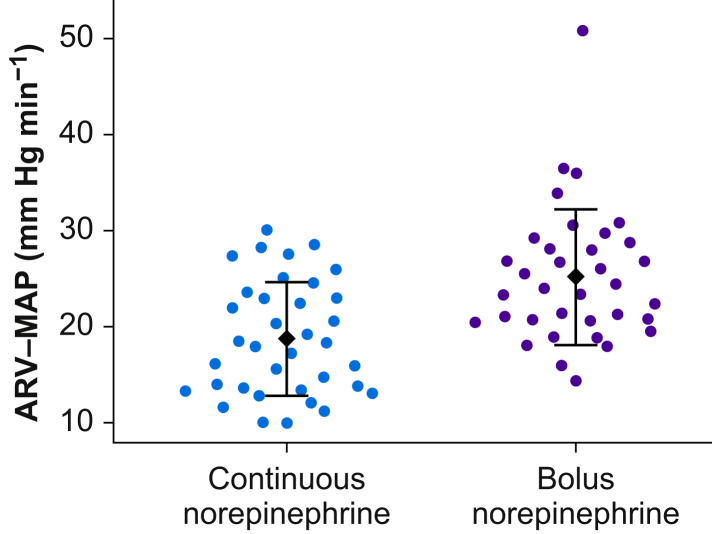
Generalised average real variability of mean arterial pressure (ARV-MAP). Means (black ♦) (standard deviation; error bars) are shown, with overlaying scatterplots in patients assigned to continuous norepinephrine infusion and manual bolus norepinephrine administration during induction of general anaesthesia.

The mean (standard deviation) generalised ARV-MAP was 19 (6) mm Hg min^−1^ in participants assigned to continuous norepinephrine infusion, compared with 25 (7) mm Hg min^−1^ in participants assigned to manual bolus norepinephrine administration (Cohen’s *d*=1.00; *P*<0.001) (Fig. 2).

### Secondary outcomes

**Fig 3:**
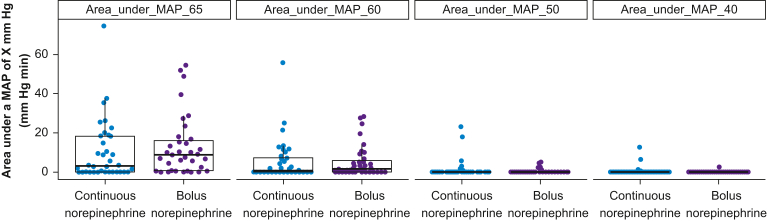
Areas under mean arterial pressure (MAP) values of 65, 60, 50, and 40 mm Hg in patients assigned to continuous norepinephrine infusion or manual bolus norepinephrine administration during induction of general anaesthesia. Boxplots with overlaying scatterplots are shown.

The median (25th, 75th percentile) area under a MAP of 65 mm Hg was 3 (0, 18) mm Hg min in participants assigned to continuous norepinephrine infusion and 9 (1, 16) mm Hg min in participants assigned to manual bolus norepinephrine administration (Cliff’s delta: 0.19; *P*=0.178, Table 2, Fig. 3). The median cumulative duration of MAP values less than 65 mm Hg was 0.8 (0.0, 2.4) min in participants assigned to continuous norepinephrine infusion and 1.8 (0.5, 2.8) min in participants assigned to manual bolus norepinephrine administration (Cliff’s delta: 0.30; *P*=0.027).

**Table 2:** Secondary endpoints quantifying hypotension. Data are presented as median (25th percentile, 75th percentile). MAP, mean arterial pressure. ∗*P*-values correspond to Wilcoxon rank-sum tests with continuity correction.

Outcome	Continuous norepinephrine infusion	Manual bolus norepinephrine administration	*P*-value∗	Cliff’s delta
	(*n*=36)	(*n*=35)		
Area under a MAP of 65 mm Hg (mm Hg min)	3 (0, 18)	9 (1, 16)	0.178	0.19
Area under a MAP of 60 mm Hg (mm Hg min)	1 (0, 7)	2 (0, 6)	0.659	0.06
Area under a MAP of 50 mm Hg (mm Hg min)	0 (0, 0)	0 (0, 0)	0.465	−0.08
Area under a MAP of 40 mm Hg (mm Hg min)	0 (0, 0)	0 (0, 0)	0.182	−0.08
Cumulative duration of MAP values <65 mm Hg, min	0.8 (0.0, 2.4)	1.8 (0.5, 2.8)	0.027	0.30
Cumulative duration of MAP values <60 mm Hg, min	0.3 (0.0, 1.4)	0.7 (0.0, 1.5)	0.311	0.14
Cumulative duration of MAP values <50 mm Hg, min	0.0 (0.0, 0.2)	0.0 (0.0, 0.0)	0.534	−0.07
Cumulative duration of MAP values <40 mm Hg, min	0.0 (0.0, 0.0)	0.0 (0.0, 0.0)	0.169	−0.08

The median area above a MAP of 100 mm Hg was 20 (2, 72) mm Hg min in participants assigned to continuous norepinephrine infusion and 16 (6, 53) mm Hg min in participants assigned to manual bolus norepinephrine administration (Cliff’s delta: −0.003; *P*=0.986) (Table 3).

**Table 3:** Secondary endpoints quantifying hypertension. Data are presented as median (25th percentile, 75th percentile). MAP, mean arterial pressure. ∗*P*-values correspond to Wilcoxon rank-sum tests with continuity correction.

Outcome	Continuous norepinephrine infusion	Manual bolus norepinephrine administration	*P*-value∗	Cliff’s delta
	(*n*=36)	(*n*=35)		
Area above a MAP of 100 mm Hg (mm Hg min)	20 (2, 72)	16 (6, 53)	0.986	−0.003
Area above a MAP of 110 mm Hg (mm Hg min)	1 (0, 24)	2 (0, 19)	0.834	0.03
Area above a MAP of 120 mm Hg (mm Hg min)	0 (0, 1)	0 (0, 4)	0.454	0.09
Area above a MAP of 140 mm Hg (mm Hg min)	0 (0, 0)	0 (0, 0)	0.274	0.09
Cumulative duration of MAP values >100 mm Hg (min)	2.8 (0.6, 5.0)	2.3 (1.3, 4.3)	0.530	−0.09
Cumulative duration of MAP values >110 mm Hg (min)	0.6 (0.0, 3.2)	0.7 (0.0, 2.2)	0.701	−0.05
Cumulative duration of MAP values >120 mm Hg (min)	0.0 (0.0, 0.6)	0.0 (0.0, 0.6)	0.691	0.05
Cumulative duration of MAP values >140 mm Hg (min)	0.0 (0.0, 0.0)	0.0 (0.0, 0.0)	0.244	0.09

The median cumulative dose of norepinephrine was 1.3 (1.0, 1.7) μg kg^−1^ in participants assigned to continuous norepinephrine infusion and 0.5 (0.4, 0.7) μg kg^−1^ in participants assigned to manual bolus norepinephrine administration (Cliff’s delta: −0.87, *P*<0.001). *Post hoc* analyses for SAP are reported in Supplementary Table S1, and analyses related to cardiac index and systemic vascular resistance index are presented in Supplementary Figures S1–S4.

## Discussion

Giving norepinephrine continuously during induction of general anaesthesia—compared with giving it as repeated manual boluses—improved blood pressure stability in high-risk noncardiac surgery patients who had continuous blood pressure monitoring with an arterial catheter. Specifically, patients assigned to continuous norepinephrine infusion had a 25% lower generalised ARV-MAP than patients assigned to manual bolus norepinephrine administration, indicating improved MAP stability during induction of general anaesthesia. Continuous norepinephrine infusion thus provided more consistent haemodynamic control during induction of general anaesthesia in high-risk patients.

Our primary endpoint was blood pressure stability during induction of general anaesthesia, quantified as generalised ARV-MAP.^13^ Generalised ARV-MAP quantifies MAP changes over time by measuring differences between consecutive MAP values. The generalised ARV-MAP was 19 mm Hg min^−1^ in patients assigned to continuous norepinephrine infusion and 25 mm Hg min^−1^ in patients assigned to manual bolus norepinephrine administration—a reduction that was statistically significant. A generalised ARV-MAP of 25 mm Hg min^−1^ means that, on average, there was a total of a 25-mm Hg MAP change within each minute (i.e. over six 10-s epochs) of the 15-min observation period. Notably, ARV-MAP does not indicate whether MAP values crossed specific thresholds, such as a MAP of 65 mm Hg, and should not be interpreted as a direct measure of hypotension. Whether improved blood pressure stability translates into less postinduction hypotension and clinically meaningful benefits remains to be determined.

As secondary endpoints, we quantified postinduction hypotension. In patients assigned to continuous norepinephrine infusion, the median cumulative duration of MAP values less than 65 mm Hg was half that observed in patients assigned to manual bolus norepinephrine administration. Additionally, the area under a MAP of 65 mm Hg was reduced by about two-thirds in the continuous *vs* bolus group, although non-significantly. There was no meaningful difference between cardiac index and systemic vascular resistance index values between the groups, and generalised ARV of these two haemodynamic variables also did not differ.

The findings of the present trial complement those of a previous trial, in which continuous—compared with intermittent—norepinephrine administration did not reduce postinduction hypotension in low-to-moderate risk noncardiac surgery patients who had intermittent oscillometric blood pressure monitoring.^14^ In contrast, in the present trial, we included high-risk patients who all had continuous blood pressure monitoring with an arterial catheter. The median age of our patients was 64 yr. Half of the patients had chronic arterial hypertension, and nearly half were classified as ASA status 3, which underscores their high-risk profile. Age ≥50 yr, chronic arterial hypertension, and ASA physical status ≥3 are risk factors for postinduction hypotension.^3^^,^^4^ Older age and chronic hypertension increase the risk for blood pressure instability owing to diminished vascular elasticity and autonomic regulation,^15^ potentially exacerbating hypotensive episodes during induction of general anaesthesia. Similarly, higher ASA physical status reflects a greater comorbidity burden,^16^ often correlating with compromised cardiovascular function.

In a previous trial, we showed that continuous intraarterial blood pressure monitoring during induction of general anaesthesia reduced the area under a MAP of 65 mm Hg from 46 to 15 mm Hg min in noncardiac surgery patients.^8^ Consequently, we now routinely insert arterial catheters before starting induction of general anaesthesia in high-risk patients with an indication for intraarterial blood pressure monitoring.^9^ In our present trial, the area under a MAP of 65 mm Hg was lower than in our previous trial (even in patients assigned to manual bolus norepinephrine administration), but continuous norepinephrine infusion during induction of general anaesthesia further reduced the median area under a MAP of 65 mm Hg—from 9 to 3 mm Hg min.

The cumulative dose of norepinephrine was significantly higher in patients assigned to continuous norepinephrine infusion than in patients assigned to manual bolus norepinephrine administration. Importantly, continuous norepinephrine infusion did not provoke hypertension, as reflected by small areas above MAP values of 100, 110, 120, and 140 mm Hg and short cumulative durations of MAP values higher than 100, 110, 120, and 140 mm Hg. Previous studies have reported associations between higher doses of norepinephrine and an increased risk of acute kidney injury.^17^^,^^18^ Whether this association reflects a causal relationship remains unclear and needs to be investigated in trials.

Limitations of our trial include the small sample size in one centre and the exclusive focus on blood pressure stability within 15 min after starting induction of general anaesthesia but not throughout surgery. In our centre, we routinely administer norepinephrine as a continuous infusion during surgery to treat intraoperative hypotension, as is similar across Europe.^19^ However, the choice of vasopressors may vary across regions and institutions, and results might vary with other vasopressors. Furthermore, our trial was not designed to determine whether the observed difference in generalised ARV-MAP is clinically meaningful. It would require a large trial to assess whether improved blood pressure stability translates into better patient-centred outcomes. Additionally, clinicians were aware of the group allocation, which could have influenced haemodynamic management. Induction of general anaesthesia was not standardised, and the type and dose of anaesthetic agents were left to the discretion of the attending clinicians. Although types and doses of anaesthetic agents were similar between groups, depth of anaesthesia was not systematically monitored and could have influenced blood pressure during induction of general anaesthesia. Finally, we excluded patients who had clear clinical indications for continuous norepinephrine infusion during induction of general anaesthesia, including those with severe aortic valve stenosis or cardiac dysfunction.

In conclusion, giving norepinephrine continuously during induction of general anaesthesia—compared with giving it as repeated manual boluses—improved blood pressure stability in high-risk noncardiac surgery patients who had continuous blood pressure monitoring with an arterial catheter. In such patients, clinicians might consider giving norepinephrine continuously to improve blood pressure stability during induction of general anaesthesia—keeping in mind that whether improved haemodynamic stability during induction improves patient-centred outcomes remains to be determined.

## Authors’ contributions

Study conception/design: CV, KK, BS, KKT

Study measurements: CV, AK

Data analysis/interpretation: all authors

Statistical analysis: CV, KKT, LK, BS

Drafting of manuscript: DIS, BS, KKT

Critical revision of article for important intellectual content: all authors

Final approval of the version to be published: all authors

Agreement to be accountable for all aspects of the work thereby ensuring that questions related to the accuracy or integrity of any part of the work are appropriately investigated and resolved: all authors

CV, LK, and KKT had full access to all trial data and are responsible for the integrity of the data and the accuracy of the data analysis.

## Funding

Support was provided solely from institutional and departmental sources.

## Declarations of interest

KK is a consultant for and has received honoraria for giving lectures from Edwards Lifesciences (Irvine, CA, USA). KK is a consultant for Vygon (Aachen, Germany). MF is a consultant for and has received honoraria for giving lectures from Edwards Lifesciences (Irvine, CA, USA). MF has received honoraria for consulting and giving lectures from CNSystems Medizintechnik (Graz, Austria). DIS is a consultant for Perceptive Medical (Newport Beach, CA, USA) and Dynacardia (Cambridge, MA, USA). BS is a consultant for Edwards Lifesciences (Irvine, CA, USA), Philips North America (Cambridge, MA, USA), GE Healthcare (Chicago, IL, USA), Maquet Critical Care (Solna, Sweden), Pulsion Medical Systems (Feldkirchen, Germany), Vygon (Aachen, Germany), Retia Medical (Valhalla, NY, USA), Masimo (Neuchâtel, Switzerland), Dynocardia (Cambridge, MA, USA). BS has received institutional restricted research grants from Edwards Lifesciences, Baxter (Deerfield, IL, USA), GE Healthcare, CNSystems Medizintechnik (Graz, Austria), Pulsion Medical Systems, Vygon, Retia Medical, Osypka Medical (Berlin, Germany). BS has received honoraria for giving lectures from Edwards Lifesciences, Philips Medizin Systeme Böblingen (Böblingen, Germany), Baxter, GE Healthcare, CNSystems Medizintechnik, Getinge (Gothenburg, Sweden), Pulsion Medical Systems, Vygon, Masimo, Ratiopharm (Ulm, Germany). BS is an Editor of the *British Journal of Anaesthesia*. KKT has received honoraria for consulting and for giving lectures from Masimo (Neuchâtel, Switzerland). The other authors declare that they have no conflicts of interest.
