# Supplementary material for: Continuous versus bolus norepinephrine administration and arterial blood pressure stability during induction of general anaesthesia in high-risk noncardiac surgery patients: a randomised trial
Source: Br J Anaesth. 2025 Jul 30;135(4):878–85. doi: 10.1016/j.bja.2025.06.025 (PMC12674030; doi:10.1016/j.bja.2025.06.025)

Supplementary Table S1: Data on systolic arterial pressure

Outcome	Continuous norepinephrine infusion (n=36)	Manual bolus norepinephrine administration (n=35)	P-values	Cohen´s d/ Cliff's delta
Generalised ARV-SAP, mmHg/min	27 (9)	36 (8)	<0.001[a]	1.07[c]
Area under a SAP of 90 mmHg, mmHg x min	1 (0, 10)	3 (0, 17)	0.375[b]	-0.12[d]
Area under a SAP of 80 mmHg, mmHg x min	0 (0, 3)	0 (0, 3)	0.659[b]	-0.05[d]
Area under a SAP of 70 mmHg, mmHg x min	0 (0, 0)	0 (0, 0)	0.212[b]	0.12[d]
Cumulative duration of SAP values <90 mmHg, min	0.2 (0.0, 1.0)	0.8 (0.0, 2.6)	0.136[b]	-0.20[d]
Cumulative duration of SAP values <80 mmHg, min	0.0 (0.0, 0.3)	0.0 (0.0, 0.8)	0.469[b]	-0.09[d]
Cumulative duration of SAP values <70 mmHg, min	0.0 (0.0, 0.2)	0.0 (0.0, 0.0)	0.214[b]	0.12[d]

Data are presented as median (25th percentile, 75th percentile) or mean (standard deviation). ARV, average real variability; SAP, systolic arterial pressure.

[a]P values correspond to a two-sample two-sided t-test.

[b]P values correspond to Wilcoxon rank-sum tests with continuity correction.

[c] corresponds to Cohen´s d.

[d] corresponds to Cliff´s delta.

Supplementary Figure S1: Boxplots illustrating cardiac index (CI) every minute during induction of general anaesthesia in patients assigned to continuous norepinephrine infusion and manual bolus norepinephrine administration.

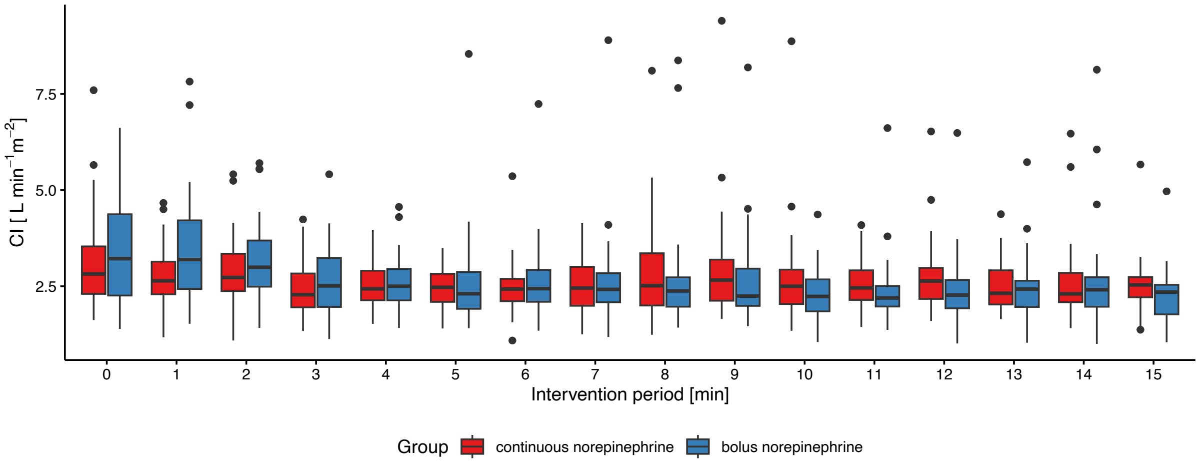

Supplementary Figure S2: Boxplots illustrating systemic vascular resistance index (SVRI) every minute during induction of general anaesthesia in patients assigned to continuous norepinephrine infusion and manual bolus norepinephrine administration.

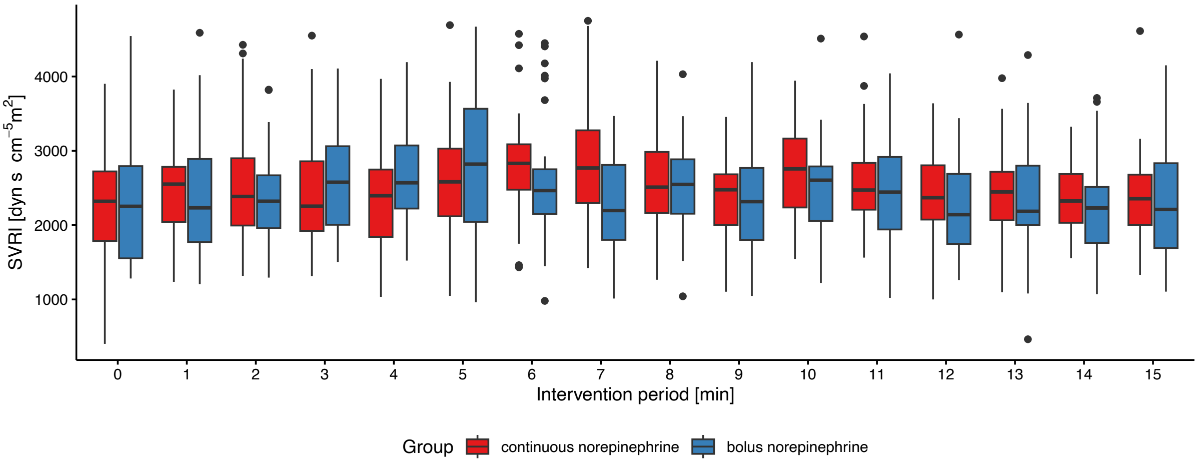

Supplementary Figure S3: Mean (black ♦) ± standard deviation (error bars) generalised average real variability of cardiac index (ARV-CI) with overlaying scatter plots in patients assigned to continuous norepinephrine infusion and (1.4 ± 0.5 L min^{-1} m^{-2}/min) and manual bolus norepinephrine administration (1.4 ± 0.5 L min^{-1} m^{-2}/min) during induction of general anaesthesia (Cohen's d: -0.09; p=0.706).

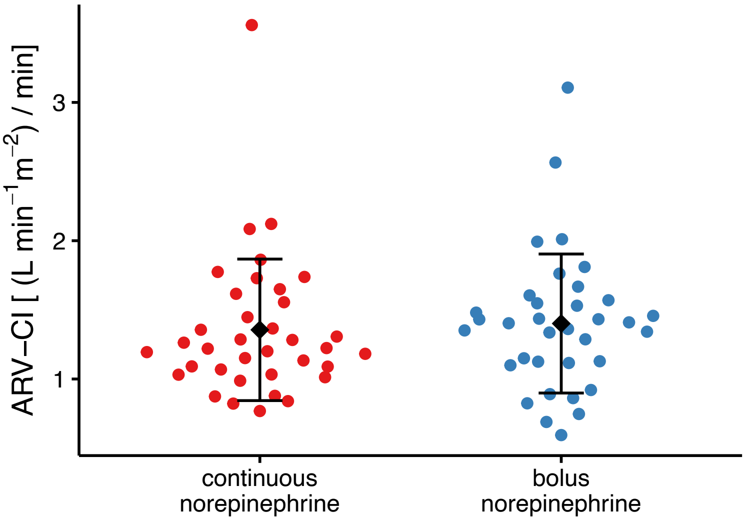

Supplementary Figure S4: Mean (black ♦) ± standard deviation (error bars) generalised average real variability of systemic vascular resistance index (ARV-SVRI) with overlaying scatter plots in patients assigned to continuous norepinephrine infusion (1158 ± 397 dyn s cm^{-5} m^{2}/min) and manual bolus norepinephrine administration (1200 ± 318 dyn s cm^{-5} m^{2}/min) during induction of general anaesthesia (Cohen's d: -0.12; p=0.624).

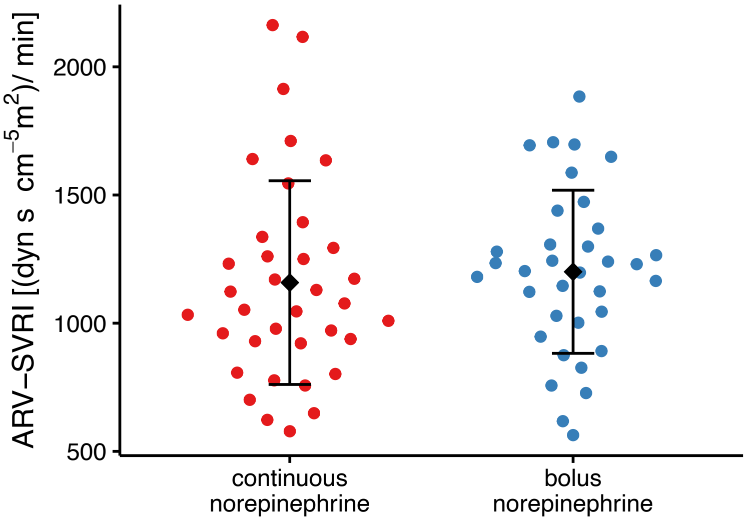

Supplement: Multimedia component 1 [file mmc1.pdf]
